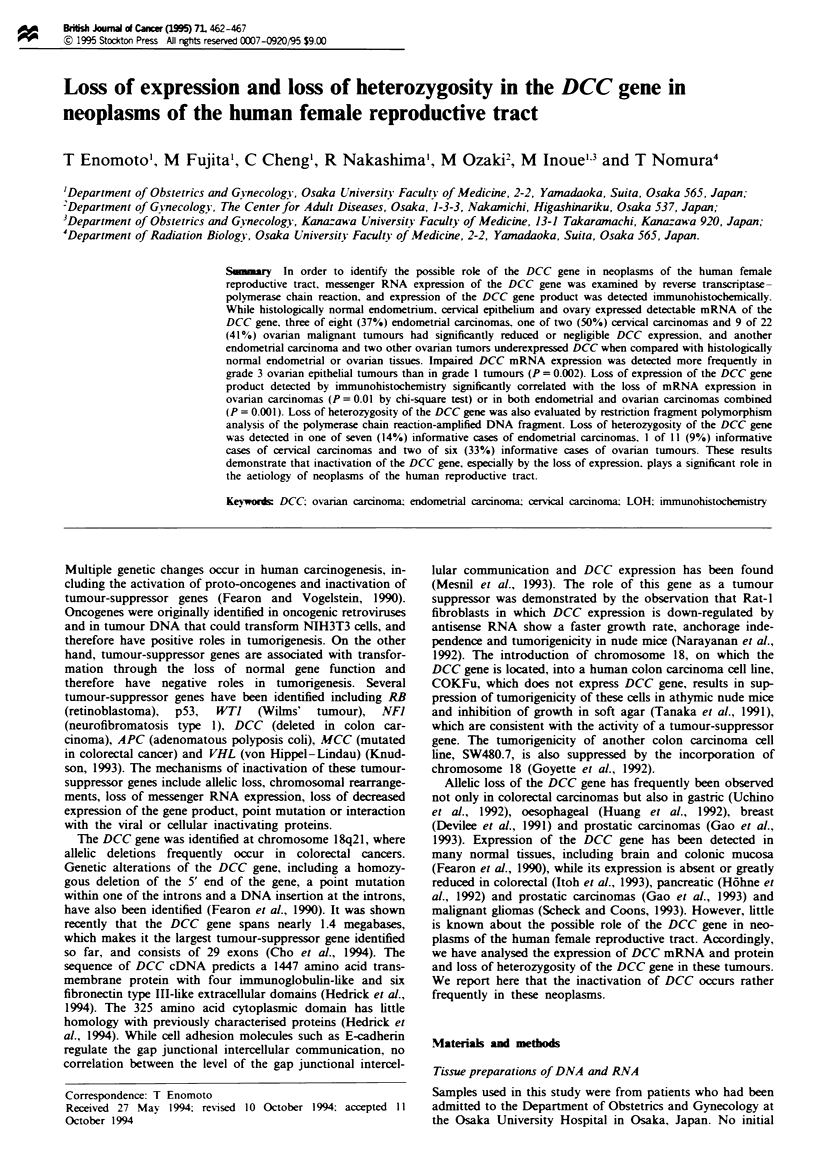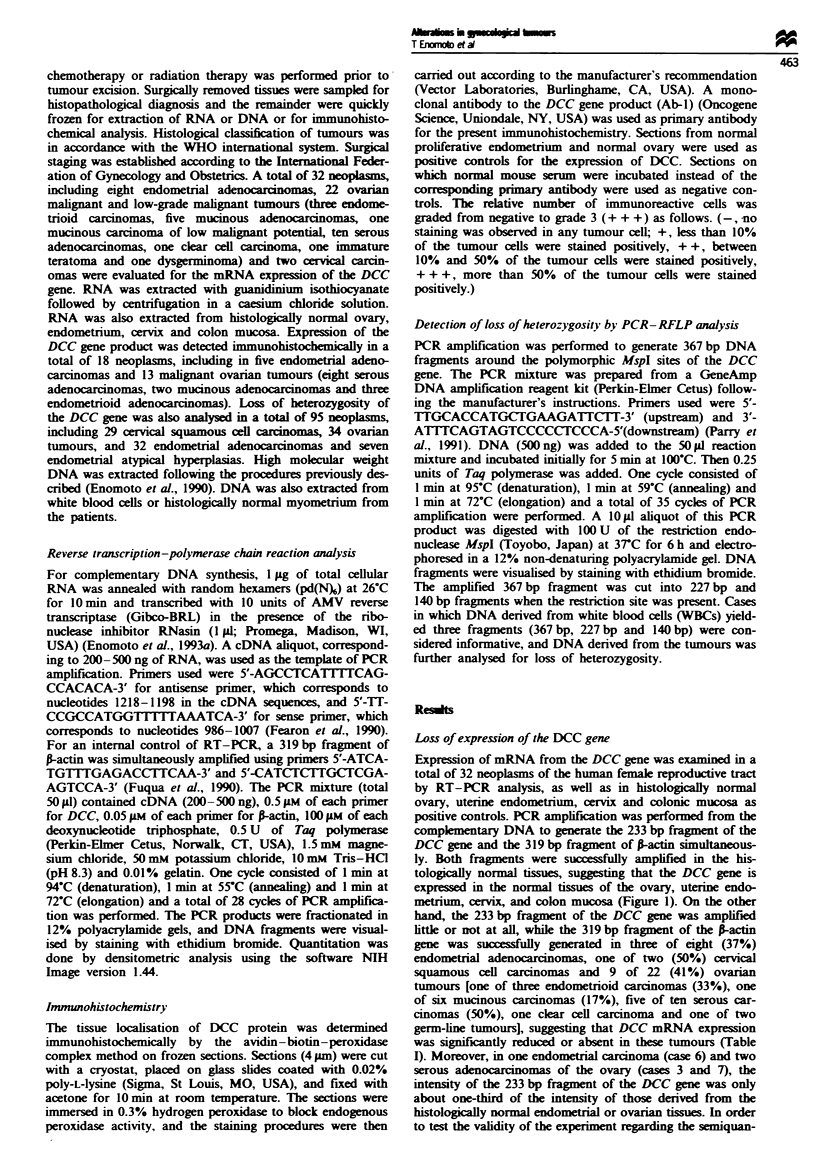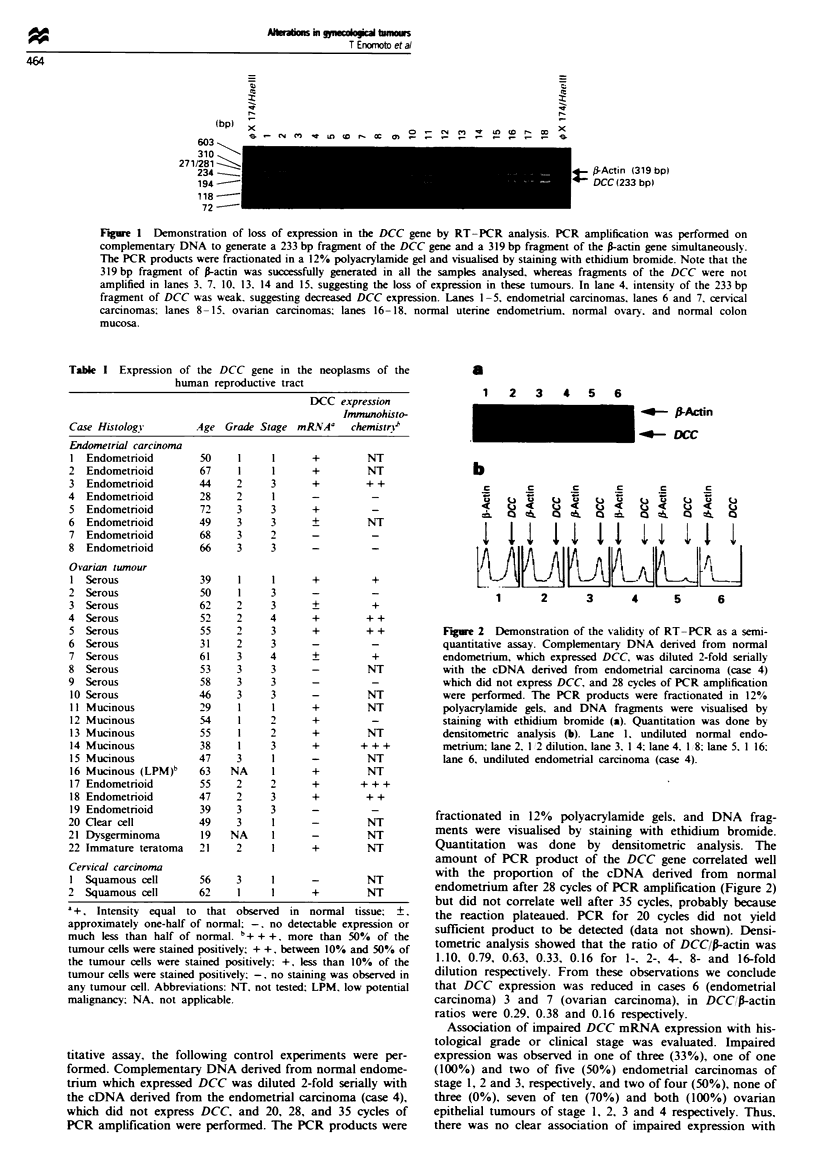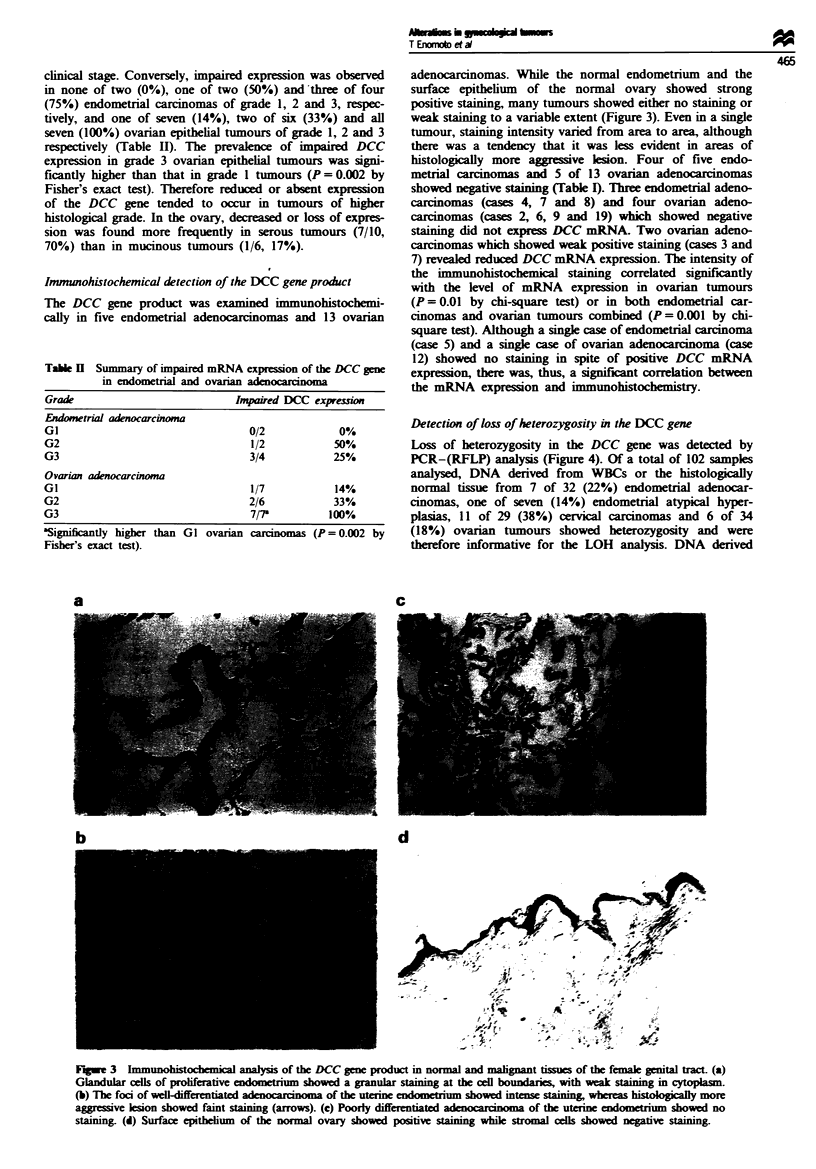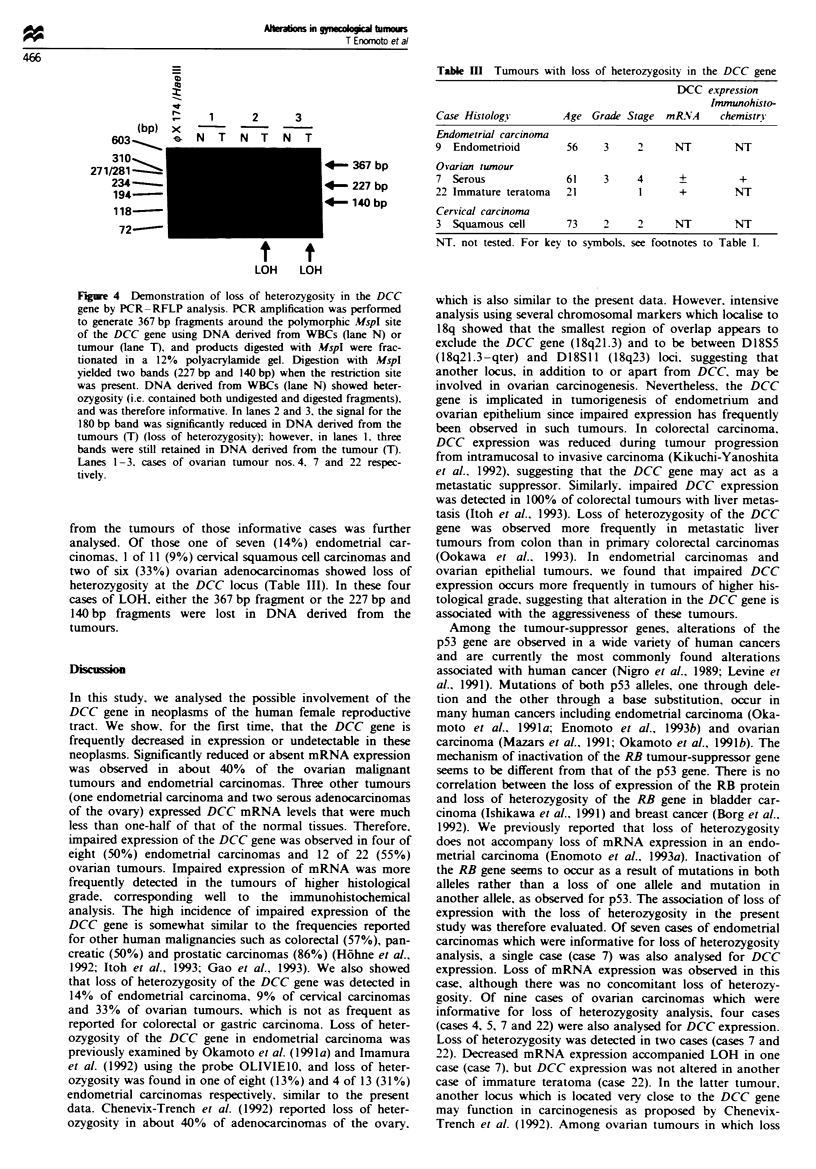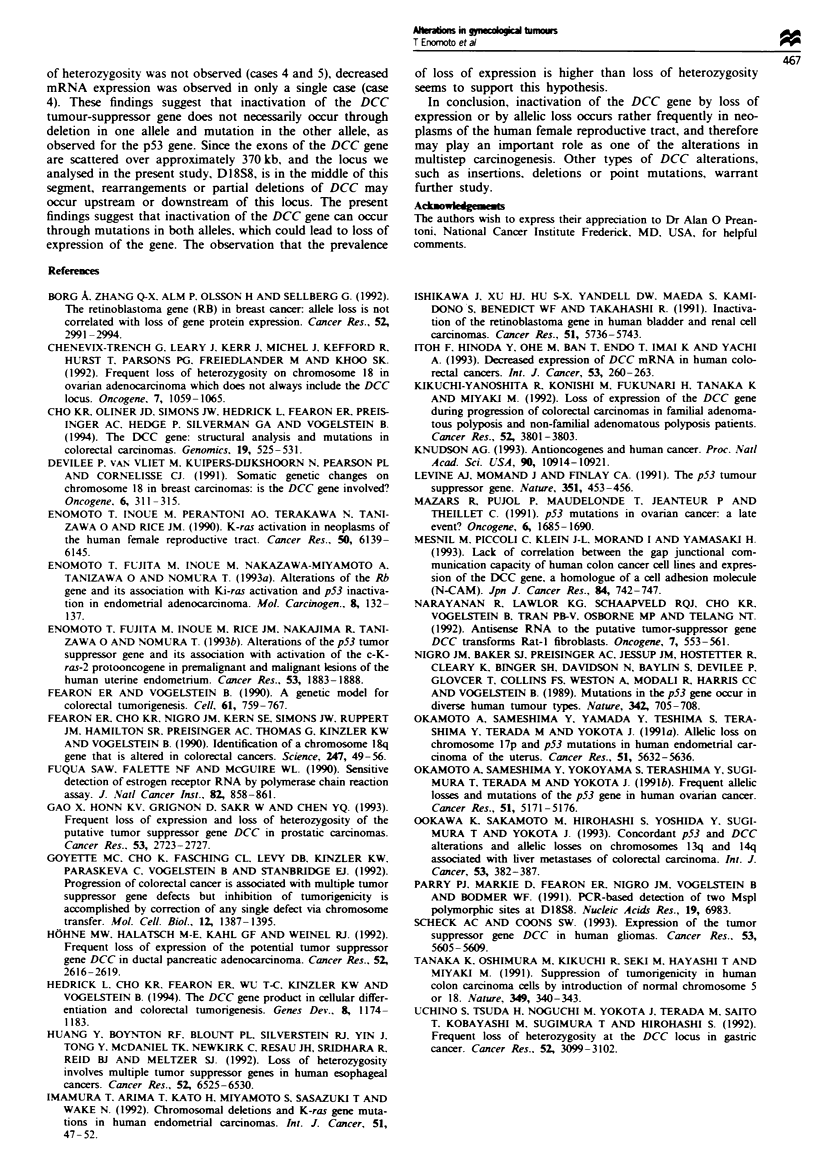# Loss of expression and loss of heterozygosity in the DCC gene in neoplasms of the human female reproductive tract.

**DOI:** 10.1038/bjc.1995.94

**Published:** 1995-03

**Authors:** T. Enomoto, M. Fujita, C. Cheng, R. Nakashima, M. Ozaki, M. Inoue, T. Nomura

**Affiliations:** Department of Obstetrics and Gynecology, Osaka University Faculty of Medicine, Japan.

## Abstract

**Images:**


British Journal of Cancer (1995) 71, 462-467

© 1995 Stockton Press All rights reserved 0007-0920/95 $9.00

### Loss of expression and loss of heterozygosity in the *DCC* gene in neoplasms of the human female reproductive tract

T Enomoto[1], M Fujita[1], C Cheng[1], R Nakashima[1], M Ozaki[2], M Inoue[1,3] and T Nomura[4]

[1]*Department of Obstetrics and Gynecology, Osaka University Faculty of Medicine, 2-2, Yamadaoka, Suita, Osaka 565, Japan;* [2]*Department of Gynecology, The Center for Adult Diseases, Osaka, 1-3-3, Nakamichi, Higashinariku, Osaka 537, Japan;* [3]*Department of Obstetrics and Gynecology, Kanazawa University Faculty of Medicine, 13-1 Takaramachi, Kanazawa 920, Japan;* [4]*Department of Radiation Biology, Osaka University Faculty of Medicine, 2-2, Yamadaoka, Suita, Osaka 565, Japan.*

**Summary** In order to identify the possible role of the *DCC* gene in neoplasms of the human female reproductive tract, messenger RNA expression of the *DCC* gene was examined by reverse transcriptase-polymerase chain reaction, and expression of the *DCC* gene product was detected immunohistochemically. While histologically normal endometrium, cervical epithelium and ovary expressed detectable mRNA of the *DCC* gene, three of eight (37%) endometrial carcinomas, one of two (50%) cervical carcinomas and 9 of 22 (41%) ovarian malignant tumours had significantly reduced or negligible *DCC* expression, and another endometrial carcinoma and two other ovarian tumors underexpressed *DCC* when compared with histologically normal endometrial or ovarian tissues. Impaired *DCC* mRNA expression was detected more frequently in grade 3 ovarian epithelial tumours than in grade 1 tumours ($P = 0.002$). Loss of expression of the *DCC* gene product detected by immunohistochemistry significantly correlated with the loss of mRNA expression in ovarian carcinomas ($P = 0.01$ by chi-square test) or in both endometrial and ovarian carcinomas combined ($P = 0.001$). Loss of heterozygosity of the *DCC* gene was also evaluated by restriction fragment polymorphism analysis of the polymerase chain reaction-amplified DNA fragment. Loss of heterozygosity of the *DCC* gene was detected in one of seven (14%) informative cases of endometrial carcinomas, 1 of 11 (9%) informative cases of cervical carcinomas and two of six (33%) informative cases of ovarian tumours. These results demonstrate that inactivation of the *DCC* gene, especially by the loss of expression, plays a significant role in the aetiology of neoplasms of the human reproductive tract.

**Keywords:** *DCC*; ovarian carcinoma; endometrial carcinoma; cervical carcinoma; LOH; immunohistochemistry

Multiple genetic changes occur in human carcinogenesis, including the activation of proto-oncogenes and inactivation of tumour-suppressor genes (Fearon and Vogelstein, 1990). Oncogenes were originally identified in oncogenic retroviruses and in tumour DNA that could transform NIH3T3 cells, and therefore have positive roles in tumorigenesis. On the other hand, tumour-suppressor genes are associated with transformation through the loss of normal gene function and therefore have negative roles in tumorigenesis. Several tumour-suppressor genes have been identified including *RB* (retinoblastoma), p53, *WT1* (Wilms' tumour), *NF1* (neurofibromatosis type 1), *DCC* (deleted in colon carcinoma), *APC* (adenomatous polyposis coli), *MCC* (mutated in colorectal cancer) and *VHL* (von Hippel-Lindau) (Knudson, 1993). The mechanisms of inactivation of these tumour-suppressor genes include allelic loss, chromosomal rearrangements, loss of messenger RNA expression, loss of decreased expression of the gene product, point mutation or interaction with the viral or cellular inactivating proteins.

The *DCC* gene was identified at chromosome 18q21, where allelic deletions frequently occur in colorectal cancers. Genetic alterations of the *DCC* gene, including a homozygous deletion of the 5′ end of the gene, a point mutation within one of the introns and a DNA insertion at the introns, have also been identified (Fearon *et al.*, 1990). It was shown recently that the *DCC* gene spans nearly 1.4 megabases, which makes it the largest tumour-suppressor gene identified so far, and consists of 29 exons (Cho *et al.*, 1994). The sequence of *DCC* cDNA predicts a 1447 amino acid transmembrane protein with four immunoglobulin-like and six fibronectin type III-like extracellular domains (Hedrick *et al.*, 1994). The 325 amino acid cytoplasmic domain has little homology with previously characterised proteins (Hedrick *et al.*, 1994). While cell adhesion molecules such as E-cadherin regulate the gap junctional intercellular communication, no correlation between the level of the gap junctional intercellular communication and *DCC* expression has been found (Mesnil *et al.*, 1993). The role of this gene as a tumour suppressor was demonstrated by the observation that Rat-1 fibroblasts in which *DCC* expression is down-regulated by antisense RNA show a faster growth rate, anchorage independence and tumorigenicity in nude mice (Narayanan *et al.*, 1992). The introduction of chromosome 18, on which the *DCC* gene is located, into a human colon carcinoma cell line, COKFu, which does not express *DCC* gene, results in suppression of tumorigenicity of these cells in athymic nude mice and inhibition of growth in soft agar (Tanaka *et al.*, 1991), which are consistent with the activity of a tumour-suppressor gene. The tumorigenicity of another colon carcinoma cell line, SW480.7, is also suppressed by the incorporation of chromosome 18 (Goyette *et al.*, 1992).

Allelic loss of the *DCC* gene has frequently been observed not only in colorectal carcinomas but also in gastric (Uchino *et al.*, 1992), oesophageal (Huang *et al.*, 1992), breast (Devilee *et al.*, 1991) and prostatic carcinomas (Gao *et al.*, 1993). Expression of the *DCC* gene has been detected in many normal tissues, including brain and colonic mucosa (Fearon *et al.*, 1990), while its expression is absent or greatly reduced in colorectal (Itoh *et al.*, 1993), pancreatic (Höhne *et al.*, 1992) and prostatic carcinomas (Gao *et al.*, 1993) and malignant gliomas (Scheck and Coons, 1993). However, little is known about the possible role of the *DCC* gene in neoplasms of the human female reproductive tract. Accordingly, we have analysed the expression of *DCC* mRNA and protein and loss of heterozygosity of the *DCC* gene in these tumours. We report here that the inactivation of *DCC* occurs rather frequently in these neoplasms.

### Materials and methods

### *Tissue preparations of DNA and RNA*

Samples used in this study were from patients who had been admitted to the Department of Obstetrics and Gynecology at the Osaka University Hospital in Osaka, Japan. No initial

Correspondence: T Enomoto
Received 27 May 1994; revised 10 October 1994; accepted 11 October 1994

chemotherapy or radiation therapy was performed prior to tumour excision. Surgically removed tissues were sampled for histopathological diagnosis and the remainder were quickly frozen for extraction of RNA or DNA or for immunohistochemical analysis. Histological classification of tumours was in accordance with the WHO international system. Surgical staging was established according to the International Federation of Gynecology and Obstetrics. A total of 32 neoplasms, including eight endometrial adenocarcinomas, 22 ovarian malignant and low-grade malignant tumours (three endometrioid carcinomas, five mucinous adenocarcinomas, one mucinous carcinoma of low malignant potential, ten serous adenocarcinomas, one clear cell carcinoma, one immature teratoma and one dysgerminoma) and two cervical carcinomas were evaluated for the mRNA expression of the *DCC* gene. RNA was extracted with guanidinium isothiocyanate followed by centrifugation in a caesium chloride solution. RNA was also extracted from histologically normal ovary, endometrium, cervix and colon mucosa. Expression of the *DCC* gene product was detected immunohistochemically in a total of 18 neoplasms, including in five endometrial adenocarcinomas and 13 malignant ovarian tumours (eight serous adenocarcinomas, two mucinous adenocarcinomas and three endometrioid adenocarcinomas). Loss of heterozygosity of the *DCC* gene was also analysed in a total of 95 neoplasms, including 29 cervical squamous cell carcinomas, 34 ovarian tumours, and 32 endometrial adenocarcinomas and seven endometrial atypical hyperplasias. High molecular weight DNA was extracted following the procedures previously described (Enomoto *et al.*, 1990). DNA was also extracted from white blood cells or histologically normal myometrium from the patients.

*Reverse transcription–polymerase chain reaction analysis*

For complementary DNA synthesis, 1 μg of total cellular RNA was annealed with random hexamers ($pd(N)_6$) at 26°C for 10 min and transcribed with 10 units of AMV reverse transcriptase (Gibco-BRL) in the presence of the ribonuclease inhibitor RNasin (1 μl; Promega, Madison, WI, USA) (Enomoto *et al.*, 1993*a*). A cDNA aliquot, corresponding to 200–500 ng of RNA, was used as the template of PCR amplification. Primers used were 5′-AGCCTCATTTTCAGCCACACA-3′ for antisense primer, which corresponds to nucleotides 1218–1198 in the cDNA sequences, and 5′-TTCCGCCATGGTTTTTAAATCA-3′ for sense primer, which corresponds to nucleotides 986–1007 (Fearon *et al.*, 1990). For an internal control of RT–PCR, a 319 bp fragment of β-actin was simultaneously amplified using primers 5′-ATCATGTTTGAGACCTTCAA-3′ and 5′-CATCTCTTGCTCGAAGTCCA-3′ (Fuqua *et al.*, 1990). The PCR mixture (total 50 μl) contained cDNA (200–500 ng), 0.5 μM of each primer for *DCC*, 0.05 μM of each primer for β-actin, 100 μM of each deoxynucleotide triphosphate, 0.5 U of *Taq* polymerase (Perkin-Elmer Cetus, Norwalk, CT, USA), 1.5 mM magnesium chloride, 50 mM potassium chloride, 10 mM Tris–HCl (pH 8.3) and 0.01% gelatin. One cycle consisted of 1 min at 94°C (denaturation), 1 min at 55°C (annealing) and 1 min at 72°C (elongation) and a total of 28 cycles of PCR amplification was performed. The PCR products were fractionated in 12% polyacrylamide gels, and DNA fragments were visualised by staining with ethidium bromide. Quantitation was done by densitometric analysis using the software NIH Image version 1.44.

*Immunohistochemistry*

The tissue localisation of DCC protein was determined immunohistochemically by the avidin–biotin–peroxidase complex method on frozen sections. Sections (4 μm) were cut with a cryostat, placed on glass slides coated with 0.02% poly-L-lysine (Sigma, St Louis, MO, USA), and fixed with acetone for 10 min at room temperature. The sections were immersed in 0.3% hydrogen peroxidase to block endogenous peroxidase activity, and the staining procedures were then carried out according to the manufacturer's recommendation (Vector Laboratories, Burlinghame, CA, USA). A monoclonal antibody to the *DCC* gene product (Ab-1) (Oncogene Science, Uniondale, NY, USA) was used as primary antibody for the present immunohistochemistry. Sections from normal proliferative endometrium and normal ovary were used as positive controls for the expression of DCC. Sections on which normal mouse serum were incubated instead of the corresponding primary antibody were used as negative controls. The relative number of immunoreactive cells was graded from negative to grade 3 (+ + +) as follows. (−, no staining was observed in any tumour cell; +, less than 10% of the tumour cells were stained positively, + +, between 10% and 50% of the tumour cells were stained positively, + + +, more than 50% of the tumour cells were stained positively.)

*Detection of loss of heterozygosity by PCR–RFLP analysis*

PCR amplification was performed to generate 367 bp DNA fragments around the polymorphic *Msp*I sites of the *DCC* gene. The PCR mixture was prepared from a GeneAmp DNA amplification reagent kit (Perkin-Elmer Cetus) following the manufacturer's instructions. Primers used were 5′-TTGCACCATGCTGAAGATTCTT-3′ (upstream) and 3′-ATTTCAGTAGTCCCCCTCCCA-5′(downstream) (Parry *et al.*, 1991). DNA (500 ng) was added to the 50 μl reaction mixture and incubated initially for 5 min at 100°C. Then 0.25 units of *Taq* polymerase was added. One cycle consisted of 1 min at 95°C (denaturation), 1 min at 59°C (annealing) and 1 min at 72°C (elongation) and a total of 35 cycles of PCR amplification were performed. A 10 μl aliquot of this PCR product was digested with 100 U of the restriction endonuclease *Msp*I (Toyobo, Japan) at 37°C for 6 h and electrophoresed in a 12% non-denaturing polyacrylamide gel. DNA fragments were visualised by staining with ethidium bromide. The amplified 367 bp fragment was cut into 227 bp and 140 bp fragments when the restriction site was present. Cases in which DNA derived from white blood cells (WBCs) yielded three fragments (367 bp, 227 bp and 140 bp) were considered informative, and DNA derived from the tumours was further analysed for loss of heterozygosity.

### Results

*Loss of expression of the* DCC *gene*

Expression of mRNA from the *DCC* gene was examined in a total of 32 neoplasms of the human female reproductive tract by RT–PCR analysis, as well as in histologically normal ovary, uterine endometrium, cervix and colonic mucosa as positive controls. PCR amplification was performed from the complementary DNA to generate the 233 bp fragment of the *DCC* gene and the 319 bp fragment of β-actin simultaneously. Both fragments were successfully amplified in the histologically normal tissues, suggesting that the *DCC* gene is expressed in the normal tissues of the ovary, uterine endometrium, cervix, and colon mucosa (Figure 1). On the other hand, the 233 bp fragment of the *DCC* gene was amplified little or not at all, while the 319 bp fragment of the β-actin gene was successfully generated in three of eight (37%) endometrial adenocarcinomas, one of two (50%) cervical squamous cell carcinomas and 9 of 22 (41%) ovarian tumours [one of three endometrioid carcinomas (33%), one of six mucinous carcinomas (17%), five of ten serous carcinomas (50%), one clear cell carcinoma and one of two germ-line tumours], suggesting that *DCC* mRNA expression was significantly reduced or absent in these tumours (Table I). Moreover, in one endometrial carcinoma (case 6) and two serous adenocarcinomas of the ovary (cases 3 and 7), the intensity of the 233 bp fragment of the *DCC* gene was only about one-third of the intensity of those derived from the histologically normal endometrial or ovarian tissues. In order to test the validity of the experiment regarding the semiquan-

**Figure 1** Demonstration of loss of expression in the *DCC* gene by RT–PCR analysis. PCR amplification was performed on complementary DNA to generate a 233 bp fragment of the *DCC* gene and a 319 bp fragment of the β-actin gene simultaneously. The PCR products were fractionated in a 12% polyacrylamide gel and visualised by staining with ethidium bromide. Note that the 319 bp fragment of β-actin was successfully generated in all the samples analysed, whereas fragments of the *DCC* were not amplified in lanes 3, 7, 10, 13, 14 and 15, suggesting the loss of expression in these tumours. In lane 4, intensity of the 233 bp fragment of *DCC* was weak, suggesting decreased *DCC* expression. Lanes 1–5, endometrial carcinomas, lanes 6 and 7, cervical carcinomas; lanes 8–15, ovarian carcinomas; lanes 16–18, normal uterine endometrium, normal ovary, and normal colon mucosa.

**Table I** Expression of the *DCC* gene in the neoplasms of the human reproductive tract

| *Case Histology* | *Age* | *Grade* | *Stage* | DCC *expression* *mRNA*[a] | *Immunohistochemistry*[b] |
|---|---|---|---|---|---|
| *Endometrial carcinoma* | | | | | |
| 1 Endometrioid | 50 | 1 | 1 | + | NT |
| 2 Endometrioid | 67 | 1 | 1 | + | NT |
| 3 Endometrioid | 44 | 2 | 3 | + | + + |
| 4 Endometrioid | 28 | 2 | 1 | – | – |
| 5 Endometrioid | 72 | 3 | 3 | + | – |
| 6 Endometrioid | 49 | 3 | 3 | ± | NT |
| 7 Endometrioid | 68 | 3 | 2 | – | – |
| 8 Endometrioid | 66 | 3 | 3 | – | – |
| *Ovarian tumour* | | | | | |
| 1 Serous | 39 | 1 | 1 | + | + |
| 2 Serous | 50 | 1 | 3 | – | – |
| 3 Serous | 62 | 2 | 3 | ± | + |
| 4 Serous | 52 | 2 | 4 | + | + + |
| 5 Serous | 55 | 2 | 3 | + | + + |
| 6 Serous | 31 | 2 | 3 | – | – |
| 7 Serous | 61 | 3 | 4 | ± | + |
| 8 Serous | 53 | 3 | 3 | – | NT |
| 9 Serous | 58 | 3 | 3 | – | – |
| 10 Serous | 46 | 3 | 3 | – | NT |
| 11 Mucinous | 29 | 1 | 1 | + | NT |
| 12 Mucinous | 54 | 1 | 2 | + | – |
| 13 Mucinous | 55 | 1 | 2 | + | NT |
| 14 Mucinous | 38 | 1 | 3 | + | + + + |
| 15 Mucinous | 47 | 3 | 1 | – | NT |
| 16 Mucinous (LPM)[b] | 63 | NA | 1 | + | NT |
| 17 Endometrioid | 55 | 2 | 2 | + | + + + |
| 18 Endometrioid | 47 | 2 | 3 | + | + + |
| 19 Endometrioid | 39 | 3 | 3 | – | – |
| 20 Clear cell | 49 | 3 | 1 | – | NT |
| 21 Dysgerminoma | 19 | NA | 1 | – | NT |
| 22 Immature teratoma | 21 | 2 | 1 | + | NT |
| *Cervical carcinoma* | | | | | |
| 1 Squamous cell | 56 | 3 | 1 | – | NT |
| 2 Squamous cell | 62 | 1 | 1 | + | NT |

[a]+, Intensity equal to that observed in normal tissue; ±, approximately one-half of normal; –, no detectable expression or much less than half of normal. [b]+ + +, more than 50% of the tumour cells were stained positively; + +, between 10% and 50% of the tumour cells were stained positively; +, less than 10% of the tumour cells were stained positively; –, no staining was observed in any tumour cell. Abbreviations: NT, not tested; LPM, low potential malignancy; NA, not applicable.

**Figure 2** Demonstration of the validity of RT–PCR as a semi-quantitative assay. Complementary DNA derived from normal endometrium, which expressed *DCC*, was diluted 2-fold serially with the cDNA derived from endometrial carcinoma (case 4) which did not express *DCC*, and 28 cycles of PCR amplification were performed. The PCR products were fractionated in 12% polyacrylamide gels, and DNA fragments were visualised by staining with ethidium bromide (**a**). Quantitation was done by densitometric analysis (**b**). Lane 1, undiluted normal endometrium; lane 2, 1/2 dilution, lane 3, 1/4; lane 4, 1/8; lane 5, 1/16; lane 6, undiluted endometrial carcinoma (case 4).

titative assay, the following control experiments were performed. Complementary DNA derived from normal endometrium which expressed *DCC* was diluted 2-fold serially with the cDNA derived from the endometrial carcinoma (case 4), which did not express *DCC*, and 20, 28, and 35 cycles of PCR amplification were performed. The PCR products were fractionated in 12% polyacrylamide gels, and DNA fragments were visualised by staining with ethidium bromide. Quantitation was done by densitometric analysis. The amount of PCR product of the *DCC* gene correlated well with the proportion of the cDNA derived from normal endometrium after 28 cycles of PCR amplification (Figure 2) but did not correlate well after 35 cycles, probably because the reaction plateaued. PCR for 20 cycles did not yield sufficient product to be detected (data not shown). Densitometric analysis showed that the ratio of *DCC*/β-actin was 1.10, 0.79, 0.63, 0.33, 0.16 for 1-, 2-, 4-, 8- and 16-fold dilution respectively. From these observations we conclude that *DCC* expression was reduced in cases 6 (endometrial carcinoma) 3 and 7 (ovarian carcinoma), in *DCC*/β-actin ratios were 0.29, 0.38 and 0.16 respectively.

Association of impaired *DCC* mRNA expression with histological grade or clinical stage was evaluated. Impaired expression was observed in one of three (33%), one of one (100%) and two of five (50%) endometrial carcinomas of stage 1, 2 and 3, respectively, and two of four (50%), none of three (0%), seven of ten (70%) and both (100%) ovarian epithelial tumours of stage 1, 2, 3 and 4 respectively. Thus, there was no clear association of impaired expression with clinical stage. Conversely, impaired expression was observed in none of two (0%), one of two (50%) and three of four (75%) endometrial carcinomas of grade 1, 2 and 3, respectively, and one of seven (14%), two of six (33%) and all seven (100%) ovarian epithelial tumours of grade 1, 2 and 3 respectively (Table II). The prevalence of impaired *DCC* expression in grade 3 ovarian epithelial tumours was significantly higher than that in grade 1 tumours ($P = 0.002$ by Fisher's exact test). Therefore reduced or absent expression of the *DCC* gene tended to occur in tumours of higher histological grade. In the ovary, decreased or loss of expression was found more frequently in serous tumours (7/10, 70%) than in mucinous tumours (1/6, 17%).

*Immunohistochemical detection of the* DCC *gene product*

The *DCC* gene product was examined immunohistochemically in five endometrial adenocarcinomas and 13 ovarian adenocarcinomas. While the normal endometrium and the surface epithelium of the normal ovary showed strong positive staining, many tumours showed either no staining or weak staining to a variable extent (Figure 3). Even in a single tumour, staining intensity varied from area to area, although there was a tendency that it was less evident in areas of histologically more aggressive lesion. Four of five endometrial carcinomas and 5 of 13 ovarian adenocarcinomas showed negative staining (Table I). Three endometrial adenocarcinomas (cases 4, 7 and 8) and four ovarian adenocarcinomas (cases 2, 6, 9 and 19) which showed negative staining did not express *DCC* mRNA. Two ovarian adenocarcinomas which showed weak positive staining (cases 3 and 7) revealed reduced *DCC* mRNA expression. The intensity of the immunohistochemical staining correlated significantly with the level of mRNA expression in ovarian tumours ($P = 0.01$ by chi-square test) or in both endometrial carcinomas and ovarian tumours combined ($P = 0.001$ by chi-square test). Although a single case of endometrial carcinoma (case 5) and a single case of ovarian adenocarcinoma (case 12) showed no staining in spite of positive *DCC* mRNA expression, there was, thus, a significant correlation between the mRNA expression and immunohistochemistry.

**Table II** Summary of impaired mRNA expression of the *DCC* gene in endometrial and ovarian adenocarcinoma

| *Grade* | *Impaired* DCC *expression* | |
|---|---|---|
| *Endometrial adenocarcinoma* | | |
| G1 | 0/2 | 0% |
| G2 | 1/2 | 50% |
| G3 | 3/4 | 25% |
| *Ovarian adenocarcinoma* | | |
| G1 | 1/7 | 14% |
| G2 | 2/6 | 33% |
| G3 | 7/7[a] | 100% |

[a]Significantly higher than G1 ovarian carcinomas ($P = 0.002$ by Fisher's exact test).

*Detection of loss of heterozygosity in the* DCC *gene*

Loss of heterozygosity in the *DCC* gene was detected by PCR-(RFLP) analysis (Figure 4). Of a total of 102 samples analysed, DNA derived from WBCs or the histologically normal tissue from 7 of 32 (22%) endometrial adenocarcinomas, one of seven (14%) endometrial atypical hyperplasias, 11 of 29 (38%) cervical carcinomas and 6 of 34 (18%) ovarian tumours showed heterozygosity and were therefore informative for the LOH analysis. DNA derived

**Figure 3** Immunohistochemical analysis of the *DCC* gene product in normal and malignant tissues of the female genital tract. (**a**) Glandular cells of proliferative endometrium showed a granular staining at the cell boundaries, with weak staining in cytoplasm. (**b**) The foci of well-differentiated adenocarcinoma of the uterine endometrium showed intense staining, whereas histologically more aggressive lesion showed faint staining (arrows). (**c**) Poorly differentiated adenocarcinoma of the uterine endometrium showed no staining. (**d**) Surface epithelium of the normal ovary showed positive staining while stromal cells showed negative staining.

**Figure 4** Demonstration of loss of heterozygosity in the *DCC* gene by PCR-RFLP analysis. PCR amplification was performed to generate 367 bp fragments around the polymorphic *Msp*I site of the *DCC* gene using DNA derived from WBCs (lane N) or tumour (lane T), and products digested with *Msp*I were fractionated in a 12% polyacrylamide gel. Digestion with *Msp*I yielded two bands (227 bp and 140 bp) when the restriction site was present. DNA derived from WBCs (lane N) showed heterozygosity (i.e. contained both undigested and digested fragments), and was therefore informative. In lanes 2 and 3, the signal for the 180 bp band was significantly reduced in DNA derived from the tumours (T) (loss of heterozygosity); however, in lanes 1, three bands were still retained in DNA derived from the tumour (T). Lanes 1-3, cases of ovarian tumour nos. 4, 7 and 22 respectively.

from the tumours of those informative cases was further analysed. Of those one of seven (14%) endometrial carcinomas, 1 of 11 (9%) cervical squamous cell carcinomas and two of six (33%) ovarian adenocarcinomas showed loss of heterozygosity at the *DCC* locus (Table III). In these four cases of LOH, either the 367 bp fragment or the 227 bp and 140 bp fragments were lost in DNA derived from the tumours.

### Discussion

In this study, we analysed the possible involvement of the *DCC* gene in neoplasms of the human female reproductive tract. We show, for the first time, that the *DCC* gene is frequently decreased in expression or undetectable in these neoplasms. Significantly reduced or absent mRNA expression was observed in about 40% of the ovarian malignant tumours and endometrial carcinomas. Three other tumours (one endometrial carcinoma and two serous adenocarcinomas of the ovary) expressed *DCC* mRNA levels that were much less than one-half of that of the normal tissues. Therefore, impaired expression of the *DCC* gene was observed in four of eight (50%) endometrial carcinomas and 12 of 22 (55%) ovarian tumours. Impaired expression of mRNA was more frequently detected in the tumours of higher histological grade, corresponding well to the immunohistochemical analysis. The high incidence of impaired expression of the *DCC* gene is somewhat similar to the frequencies reported for other human malignancies such as colorectal (57%), pancreatic (50%) and prostatic carcinomas (86%) (Höhne *et al.*, 1992; Itoh *et al.*, 1993; Gao *et al.*, 1993). We also showed that loss of heterozygosity of the *DCC* gene was detected in 14% of endometrial carcinoma, 9% of cervical carcinomas and 33% of ovarian tumours, which is not as frequent as reported for colorectal or gastric carcinoma. Loss of heterozygosity of the *DCC* gene in endometrial carcinoma was previously examined by Okamoto *et al.* (1991*a*) and Imamura *et al.* (1992) using the probe OLIVIE10, and loss of heterozygosity was found in one of eight (13%) and 4 of 13 (31%) endometrial carcinomas respectively, similar to the present data. Chenevix-Trench *et al.* (1992) reported loss of heterozygosity in about 40% of adenocarcinomas of the ovary,

**Table III** Tumours with loss of heterozygosity in the *DCC* gene

| Case | Histology | Age | Grade | Stage | DCC expression mRNA | DCC expression Immunohistochemistry |
|---|---|---|---|---|---|---|
| *Endometrial carcinoma* | | | | | | |
| 9 | Endometrioid | 56 | 3 | 2 | NT | NT |
| *Ovarian tumour* | | | | | | |
| 7 | Serous | 61 | 3 | 4 | ± | + |
| 22 | Immature teratoma | 21 | | 1 | + | NT |
| *Cervical carcinoma* | | | | | | |
| 3 | Squamous cell | 73 | 2 | 2 | NT | NT |

NT, not tested. For key to symbols, see footnotes to Table I.

which is also similar to the present data. However, intensive analysis using several chromosomal markers which localise to 18q showed that the smallest region of overlap appears to exclude the *DCC* gene (18q21.3) and to be between D18S5 (18q21.3-qter) and D18S11 (18q23) loci, suggesting that another locus, in addition to or apart from *DCC*, may be involved in ovarian carcinogenesis. Nevertheless, the *DCC* gene is implicated in tumorigenesis of endometrium and ovarian epithelium since impaired expression has frequently been observed in such tumours. In colorectal carcinoma, *DCC* expression was reduced during tumour progression from intramucosal to invasive carcinoma (Kikuchi-Yanoshita *et al.*, 1992), suggesting that the *DCC* gene may act as a metastatic suppressor. Similarly, impaired *DCC* expression was detected in 100% of colorectal tumours with liver metastasis (Itoh *et al.*, 1993). Loss of heterozygosity of the *DCC* gene was observed more frequently in metastatic liver tumours from colon than in primary colorectal carcinomas (Ookawa *et al.*, 1993). In endometrial carcinomas and ovarian epithelial tumours, we found that impaired *DCC* expression occurs more frequently in tumours of higher histological grade, suggesting that alteration in the *DCC* gene is associated with the aggressiveness of these tumours.

Among the tumour-suppressor genes, alterations of the p53 gene are observed in a wide variety of human cancers and are currently the most commonly found alterations associated with human cancer (Nigro *et al.*, 1989; Levine *et al.*, 1991). Mutations of both p53 alleles, one through deletion and the other through a base substitution, occur in many human cancers including endometrial carcinoma (Okamoto *et al.*, 1991*a*; Enomoto *et al.*, 1993*b*) and ovarian carcinoma (Mazars *et al.*, 1991; Okamoto *et al.*, 1991*b*). The mechanism of inactivation of the *RB* tumour-suppressor gene seems to be different from that of the p53 gene. There is no correlation between the loss of expression of the RB protein and loss of heterozygosity of the *RB* gene in bladder carcinoma (Ishikawa *et al.*, 1991) and breast cancer (Borg *et al.*, 1992). We previously reported that loss of heterozygosity does not accompany loss of mRNA expression in an endometrial carcinoma (Enomoto *et al.*, 1993*a*). Inactivation of the *RB* gene seems to occur as a result of mutations in both alleles rather than a loss of one allele and mutation in another allele, as observed for p53. The association of loss of expression with the loss of heterozygosity in the present study was therefore evaluated. Of seven cases of endometrial carcinomas which were informative for loss of heterozygosity analysis, a single case (case 7) was also analysed for *DCC* expression. Loss of mRNA expression was observed in this case, although there was no concomitant loss of heterozygosity. Of nine cases of ovarian carcinomas which were informative for loss of heterozygosity analysis, four cases (cases 4, 5, 7 and 22) were also analysed for *DCC* expression. Loss of heterozygosity was detected in two cases (cases 7 and 22). Decreased mRNA expression accompanied LOH in one case (case 7), but *DCC* expression was not altered in another case of immature teratoma (case 22). In the latter tumour, another locus which is located very close to the *DCC* gene may function in carcinogenesis as proposed by Chenevix-Trench *et al.* (1992). Among ovarian tumours in which loss of heterozygosity was not observed (cases 4 and 5), decreased mRNA expression was observed in only a single case (case 4). These findings suggest that inactivation of the *DCC* tumour-suppressor gene does not necessarily occur through deletion in one allele and mutation in the other allele, as observed for the p53 gene. Since the exons of the *DCC* gene are scattered over approximately 370 kb, and the locus we analysed in the present study, D18S8, is in the middle of this segment, rearrangements or partial deletions of *DCC* may occur upstream or downstream of this locus. The present findings suggest that inactivation of the *DCC* gene can occur through mutations in both alleles, which could lead to loss of expression of the gene. The observation that the prevalence of loss of expression is higher than loss of heterozygosity seems to support this hypothesis.

In conclusion, inactivation of the *DCC* gene by loss of expression or by allelic loss occurs rather frequently in neoplasms of the human female reproductive tract, and therefore may play an important role as one of the alterations in multistep carcinogenesis. Other types of *DCC* alterations, such as insertions, deletions or point mutations, warrant further study.

**Acknowledgements**
The authors wish to express their appreciation to Dr Alan O Preantoni, National Cancer Institute Frederick, MD, USA, for helpful comments.

**References**

BORG Å, ZHANG Q-X, ALM P, OLSSON H AND SELLBERG G. (1992). The retinoblastoma gene (RB) in breast cancer: allele loss is not correlated with loss of gene protein expression. *Cancer Res.*, **52**, 2991–2994.

CHENEVIX-TRENCH G, LEARY J, KERR J, MICHEL J, KEFFORD R, HURST T, PARSONS PG, FREIEDLANDER M AND KHOO SK. (1992). Frequent loss of heterozygosity on chromosome 18 in ovarian adenocarcinoma which does not always include the *DCC* locus. *Oncogene*, **7**, 1059–1065.

CHO KR, OLINER JD, SIMONS JW, HEDRICK L, FEARON ER, PREISINGER AC, HEDGE P, SILVERMAN GA AND VOGELSTEIN B. (1994). The DCC gene: structural analysis and mutations in colorectal carcinomas. *Genomics*, **19**, 525–531.

DEVILEE P, VAN VLIET M, KUIPERS-DIJKSHOORN N, PEARSON PL AND CORNELISSE CJ. (1991). Somatic genetic changes on chromosome 18 in breast carcinomas: is the *DCC* gene involved? *Oncogene*, **6**, 311–315.

ENOMOTO T, INOUE M, PERANTONI AO, TERAKAWA N, TANIZAWA O AND RICE JM. (1990). K-*ras* activation in neoplasms of the human female reproductive tract. *Cancer Res.*, **50**, 6139–6145.

ENOMOTO T, FUJITA M, INOUE M, NAKAZAWA-MIYAMOTO A, TANIZAWA O AND NOMURA T. (1993*a*). Alterations of the *Rb* gene and its association with Ki-*ras* activation and *p53* inactivation in endometrial adenocarcinoma. *Mol. Carcinogen.*, **8**, 132–137.

ENOMOTO T, FUJITA M, INOUE M, RICE JM, NAKAJIMA R, TANIZAWA O AND NOMURA T. (1993*b*). Alterations of the *p53* tumor suppressor gene and its association with activation of the c-K-*ras*-2 protooncogene in premalignant and malignant lesions of the human uterine endometrium. *Cancer Res.*, **53**, 1883–1888.

FEARON ER AND VOGELSTEIN B. (1990). A genetic model for colorectal tumorigenesis. *Cell*, **61**, 759–767.

FEARON ER, CHO KR, NIGRO JM, KERN SE, SIMONS JW, RUPPERT JM, HAMILTON SR, PREISINGER AC, THOMAS G, KINZLER KW AND VOGELSTEIN B. (1990). Identification of a chromosome 18q gene that is altered in colorectal cancers. *Science*, **247**, 49–56.

FUQUA SAW, FALETTE NF AND McGUIRE WL. (1990). Sensitive detection of estrogen receptor RNA by polymerase chain reaction assay. *J. Natl Cancer Inst.*, **82**, 858–861.

GAO X, HONN KV, GRIGNON D, SAKR W AND CHEN YQ. (1993). Frequent loss of expression and loss of heterozygosity of the putative tumor suppressor gene *DCC* in prostatic carcinomas. *Cancer Res.*, **53**, 2723–2727.

GOYETTE MC, CHO K, FASCHING CL, LEVY DB, KINZLER KW, PARASKEVA C, VOGELSTEIN B AND STANBRIDGE EJ. (1992). Progression of colorectal cancer is associated with multiple tumor suppressor gene defects but inhibition of tumorigenicity is accomplished by correction of any single defect via chromosome transfer. *Mol. Cell. Biol.*, **12**, 1387–1395.

HÖHNE MW, HALATSCH M-E, KAHL GF AND WEINEL RJ. (1992). Frequent loss of expression of the potential tumor suppressor gene *DCC* in ductal pancreatic adenocarcinoma. *Cancer Res.*, **52**, 2616–2619.

HEDRICK L, CHO KR, FEARON ER, WU T-C, KINZLER KW AND VOGELSTEIN B. (1994). The *DCC* gene product in cellular differentiation and colorectal tumorigenesis. *Genes Dev.*, **8**, 1174–1183.

HUANG Y, BOYNTON RF, BLOUNT PL, SILVERSTEIN RJ, YIN J, TONG Y, McDANIEL TK, NEWKIRK C, RESAU JH, SRIDHARA R, REID BJ AND MELTZER SJ. (1992). Loss of heterozygosity involves multiple tumor suppressor genes in human esophageal cancers. *Cancer Res.*, **52**, 6525–6530.

IMAMURA T, ARIMA T, KATO H, MIYAMOTO S, SASAZUKI T AND WAKE N. (1992). Chromosomal deletions and K-*ras* gene mutations in human endometrial carcinomas. *Int. J. Cancer*, **51**, 47–52.

ISHIKAWA J, XU HJ, HU S-X, YANDELL DW, MAEDA S, KAMIDONO S, BENEDICT WF AND TAKAHASHI R. (1991). Inactivation of the retinoblastoma gene in human bladder and renal cell carcinomas. *Cancer Res.*, **51**, 5736–5743.

ITOH F, HINODA Y, OHE M, BAN T, ENDO T, IMAI K AND YACHI A. (1993). Decreased expression of *DCC* mRNA in human colorectal cancers. *Int. J. Cancer*, **53**, 260–263.

KIKUCHI-YANOSHITA R, KONISHI M, FUKUNARI H, TANAKA K AND MIYAKI M. (1992). Loss of expression of the *DCC* gene during progression of colorectal carcinomas in familial adenomatous polyposis and non-familial adenomatous polyposis patients. *Cancer Res.*, **52**, 3801–3803.

KNUDSON AG. (1993). Antioncogenes and human cancer. *Proc. Natl Acad. Sci. USA*, **90**, 10914–10921.

LEVINE AJ, MOMAND J AND FINLAY CA. (1991). The *p53* tumour suppressor gene. *Nature*, **351**, 453–456.

MAZARS R, PUJOL P, MAUDELONDE T, JEANTEUR P AND THEILLET C. (1991). *p53* mutations in ovarian cancer: a late event? *Oncogene*, **6**, 1685–1690.

MESNIL M, PICCOLI C, KLEIN J-L, MORAND I AND YAMASAKI H. (1993). Lack of correlation between the gap junctional communication capacity of human colon cancer cell lines and expression of the DCC gene, a homologue of a cell adhesion molecule (N-CAM). *Jpn J. Cancer Res.*, **84**, 742–747.

NARAYANAN R, LAWLOR KG, SCHAAPVELD RQJ, CHO KR, VOGELSTEIN B, TRAN PB-V, OSBORNE MP AND TELANG NT. (1992). Antisense RNA to the putative tumor-suppressor gene *DCC* transforms Rat-1 fibroblasts. *Oncogene*, **7**, 553–561.

NIGRO JM, BAKER SJ, PREISINGER AC, JESSUP JM, HOSTETTER R, CLEARY K, BINGER SH, DAVIDSON N, BAYLIN S, DEVILEE P, GLOVCER T, COLLINS FS, WESTON A, MODALI R, HARRIS CC AND VOGELSTEIN B. (1989). Mutations in the *p53* gene occur in diverse human tumour types. *Nature*, **342**, 705–708.

OKAMOTO A, SAMESHIMA Y, YAMADA Y, TESHIMA S, TERASHIMA Y, TERADA M AND YOKOTA J. (1991*a*). Allelic loss on chromosome 17p and *p53* mutations in human endometrial carcinoma of the uterus. *Cancer Res.*, **51**, 5632–5636.

OKAMOTO A, SAMESHIMA Y, YOKOYAMA S, TERASHIMA Y, SUGIMURA T, TERADA M AND YOKOTA J. (1991*b*). Frequent allelic losses and mutations of the *p53* gene in human ovarian cancer. *Cancer Res.*, **51**, 5171–5176.

OOKAWA K, SAKAMOTO M, HIROHASHI S, YOSHIDA Y, SUGIMURA T AND YOKOTA J. (1993). Concordant *p53* and *DCC* alterations and allelic losses on chromosomes 13q and 14q associated with liver metastases of colorectal carcinoma. *Int. J. Cancer*, **53**, 382–387.

PARRY PJ, MARKIE D, FEARON ER, NIGRO JM, VOGELSTEIN B AND BODMER WF. (1991). PCR-based detection of two MspI polymorphic sites at D18S8. *Nucleic Acids Res.*, **19**, 6983.

SCHECK AC AND COONS SW. (1993). Expression of the tumor suppressor gene *DCC* in human gliomas. *Cancer Res.*, **53**, 5605–5609.

TANAKA K, OSHIMURA M, KIKUCHI R, SEKI M, HAYASHI T AND MIYAKI M. (1991). Suppression of tumorigenicity in human colon carcinoma cells by introduction of normal chromosome 5 or 18. *Nature*, **349**, 340–343.

UCHINO S, TSUDA H, NOGUCHI M, YOKOTA J, TERADA M, SAITO T, KOBAYASHI M, SUGIMURA T AND HIROHASHI S. (1992). Frequent loss of heterozygosity at the *DCC* locus in gastric cancer. *Cancer Res.*, **52**, 3099–3102.